# Application of an artificial neural networks for predicting the heat transfer in conical spouted bed using the Nusselt module

**DOI:** 10.1016/j.heliyon.2022.e11611

**Published:** 2022-11-17

**Authors:** Juan F. Saldarriaga

**Affiliations:** Dept. Civil and Environmental Engineering, Universidad de los Andes, Carrera 1 Este #19A-40, Bogotá 111711, Colombia

**Keywords:** Heat transfer, Artificial neural networks, Conical spouted bed, Biomass

## Abstract

Artificial neural networks have been used since the last decade as a satisfactory alternative for the prediction of the fluid-dynamic behavior of particles. The aim of this work has been to develop a model based on artificial neural networks (ANN) suitable for quantifying the influence of multiple factors on the heat transfer rate in a conical spouted bed reactor. The Nusselt module has been taken as an exit point and nine input factors have been evaluated, among which are the height of the bed, the diameter of the contactor, the angle of the cone, and the minimum spouting speed, among others. The model has been found to fit appropriately to the equations proposed in the literature and can be used as a suitable model to predict the behavior of heat transfer in conical spouted bed reactors operating with biomass.

## 1 Introduction

Conical spouted bed reactors (CSBR) are a type of reactor of easy design, given that they do not require a distribution plate and have a low pressure drop [1]. The conical spouted bed reactor (CSBR) is suitable for continuous operation; in this regard, it is an alternative to the fluidized bed, which is particularly relevant for the implementation of biomass pyrolysis on a larger scale and has the additional advantage of its simple design [2]. This type of reactor has favorable features for thermal transformation processes, which are: suitable for irregular and small particles; counter-current gas-solid contact in most of the bed; and low segregation [3]. Furthermore, CSBR geometry allows operations with high gas velocities and, therefore, intense gas-solid contact, avoiding agglomeration of particles in the reactor bed [4].

CSBR has been successfully used in various thermo-physical and thermochemical operations such as pyrolysis, combustion, gasification, drying, photocatalytic degradation, coating, etc., presenting promising results at both bench and industrial-scale [5, 6, 7, 8, 9, 10, 11, 12]. The optimal performance of the thermal process in CSBR is a result of the high heat and mass transfer rates between phases favored by the countercurrent gas-solid contact [13]. For that, the transfer of heat in thermal processes is mandatory knowledge since they can be controlled both by the added gas and by the particles. Likewise, heat rates are required when materials such as biomass or plastics are added to the fluidized bed reactor, due to the need to provide heat to the processes or to remove heat from the reaction environment [14].

Different works have been reported in the literature that focus on the development of models based on mass and heat balance in order to describe heat transfer in spouted beds [15, 16, 17, 18, 19]. Others have determined heat transfer by measuring changes in the temperature of the gas passing through the bed [20]. Nevertheless, the approaches published so far require complex calculations that seek to solve these equations or are not even adequate to determine the heat transfer between the bed and the surface [14]. An alternative approach that does not have these problems involves calculating the heat transfer coefficient using the total surface area of the particles in the bed through the determination of empirical correlations for the dimensionless moduli of Nusselt [14, 19, 21, 22, 23, 24].

Nevertheless, most of these empirical correlations have been obtained using the least squares adjustment, which requires assumptions involving the structure of the equipment used and the CSBR configuration, which makes the generalization of the correlations found difficult. In addition, considering the effects of multiple influence factors in the prediction of the heat transfer or the dimensionless moduli of Nusselt is a challenging issue in empirical correlations since the models obtained are linear, yet the operation on a CSBR is non-linear [25].

Artificial neural networks (ANNs) have been demonstrated to be a capable tool to model complex non-linear multivariate problems with the advantages of learning capacity and memory simulation [26]. The ANN is a connection of processing units that are responsible for exchanging data or information, producing learning from experience, having simplified computational models of the biological structure of brain neural networks. Consequently, ANN has been considered a good option for determining the operation parameters in different configurations of CSBR [25, 27, 28]. In the literature, there are few works that have focused on the study of empirical equations that can help understand the behavior of heat transfer in conical spouted bed reactors. Therefore, the use of ANN with the help of the equations proposed by different authors could help improve this behavior. The use of ANNs has amply demonstrated that they are suitable for fitting equations with similar results, in other cases, better than empirical equations. For this reason, this work has focused on comparing the results of the ANN with the empirical equations in order to take new steps in the study of heat transfer in spouted bed reactors. The main objective of this study is to develop a model based on the (ANN) approach to quantitatively capture the influence of multiple factors on the heat transfer rate measured as the dimensionless moduli of Nusselt.

## 2 Materials and methods

### 2.1 Artificial neural network-based model

**Figure 1:**
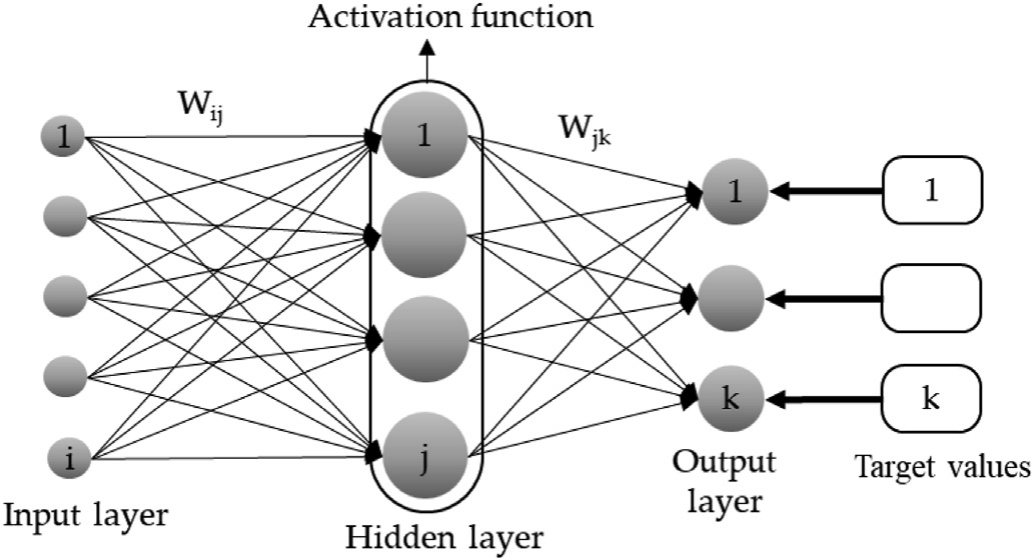
Diagram of a simplified model of an artificial neuron.

An ANN consists of different interconnected sections, which can be single or multiple layers. In ANN, input data is fed to input neurons (synapses), where these inputs are weighted by the software. The weighted sum is then operated by an activation function, and the output data is fed to other neurons in the network. All neurons are highly connected and therefore the activation values can build the result or can even feed a following model. These connection weights are modified during neuron training in order to obtain an optimal model, and the interpolation of training patterns is presented to the network during training in order to obtain the desired accuracy [29]. A simplified model of an ANN is illustrated in Figure 1.

For the present study, the multi-layer perceptron (MLP) network with backward propagation (BP) was selected since it is efficient of modeling nonlinear multivariate systems. In this network, the input layer is the one that receives the input data, and the output vector is called the output layer. Also, there are the hidden layers that do not receive any direct input and do not directly contribute to the output. The input signals propagate in a gradually modified form in the forward direction, eventually reaching the output layer. The activation function for neurons in an MLP network can be linear or non-linear with functions such as logarithmic, linear, hyperbolic tangent, sigmoidal, among others. For the analysis and adjustment of equations in this work, the herbal tangent function has been used, since in the reported studies it is the most used in an MPL configuration (Eq. (1)).

(1) $$f\left(x\right)=\frac{e^{x}-e^{-x}}{e^{x}+e^{-x}}$$

where x is the sum of all the input neurons and *f(x)* presents the range from −1 to 1.

### 2.2 Structure of ANN selected

For this study, the selected ANN structure is an MLP fully connected, consisting of an input layer, a hidden layer, and an output layer. The number of neurons in the hidden layer is determined by a trial-and-error process where the quantity and complexity of both input and output variables as well as data noise are considered. An iterative backpropagation algorithm has been implemented to determine errors for the hidden layer neurons and subsequent weight modification.

The data for training and testing was taken from the experiments executed by Saldarriaga et al. [14]. The data was extracted and formatted in accordance with the ANN requirements. The formatted data matrices [9 × 60] have 540 experimental points for the training and testing processes. To determine the best ANN model, the data was divided into training and testing data, with 60% of the total data for training and the remaining 40% for testing. To avoid any possible bias in the selection of the data, the entire dataset was divided aleatorily. The widely used Matlab 2018® software for data analysis and visualization has been used for multi-layer perceptron (MPL) training and testing processes.

### 2.3 Experimental data

The following input parameters were considered to estimate the dimensionless moduli of Nusselt: static bed height (*H*_*O*_), contactor base diameter (*D*_*i*_), probe center height (*z*), radius of the contactor (*R*), cone angle (γ), minimum spouting velocity (*u*_*ms*_), radical dimensionless position (r), Archimedes number (Ar), and particle density (ρ_b_) (Table 1). For the sawdust used in these experiments, the moisture content was determined (ISO-589 standard), using a halogen moisture analyzer (HR83, Mettler Toledo). The Archimedean number was estimated at room temperature. Finally, the density was determined by means of mercury porosimeter [30], and the average particle size (mean reciprocal diameter) was calculated from (Eq. (2)):

(2) $${\bar{d}}_{p}=\frac{1}{\sum \frac{x_{i}}{d_{p_{i}}}}$$

**Table 1:** Physical properties of sand and sawdust.

Properties	Sawdust	Sand
Mean diameter, d_p_, mm	0.77	0.17
Particle density, ρ_b_, (kg/m^3^)	495	2650
Bulk density, ρ_s_, (kg/m^3^)	188	1690
Moisture content, (wt%, d.b.)	9.1	9.3
Archimedes number, (Ar)	6.8·10^3^	4.0·10^2^
Geldart classification	D	A

### 2.4 Empirical correlation for dimensionless moduli Nusselt in CSBR

Different correlations were selected to compare with the results obtained with the neural networks. The correlations used for the determination of the dimensionless moduli of Nusselt in CSBR (Eqs. (3), (4), (5), and (6)) are listed in Table 2.

**Table 2:** Empirical correlations used.

Authors	Correlations for operating pressure drop	Eq
Kmiec [21]	$\text{Nu}=0.897Re^{0.464}Pr^{0.13}Ar^{0.116}{\left(\tan\frac{\gamma }{2}\right)}^{1.02}{\left(\frac{H_{o}}{d_{p}}\right)}^{-0.38}\varphi^{2.261}{\left(\frac{z}{H_{o}}\right)}^{1.34}$	3
Kmiec [31]	$\text{Nu}=0.045Re^{0.664}Pr^{0.333}Ar^{0.226}{\left(\tan\frac{\gamma }{2}\right)}^{-0.852}{\left(\frac{H_{o}}{d_{p}}\right)}^{-0.92}{\left(\frac{D_{i}}{d_{p}}\right)}^{1.01}\varphi^{2.304}{\left(\frac{z}{H_{o}}\right)}^{1.54}$	4
Kmiec and Kucharski [23]	$\text{Nu}=2.673Re^{0.516}Pr^{0.333}Ar^{0.033}{\left(\tan\frac{\gamma }{2}\right)}^{-2.331}{\left(\frac{H_{o}}{d_{p}}\right)}^{-1.334}{\left(\frac{D_{i}}{d_{p}}\right)}^{0.602}\varphi^{2.102}{\left(\frac{r}{R}\right)}^{-0.51}{\left(\frac{z}{H_{o}}\right)}^{4.76}$	5
Saldarriaga et al. [14]	$\text{Nu}=kRe^{0.516}Pr^{0.333}Ar^{0.033}{\left(\tan\frac{\gamma }{2}\right)}^{-2.331}{\left(\frac{H_{o}}{d_{p}}\right)}^{-1.334}{\left(\frac{D_{i}}{d_{p}}\right)}^{0.602}\varphi^{2.102}{\left(\frac{r}{R}\right)}^{-0.51}{\left(\frac{z}{H_{o}}\right)}^{4.76}$	6

## 3 Results and discussion

**Figure 2:**
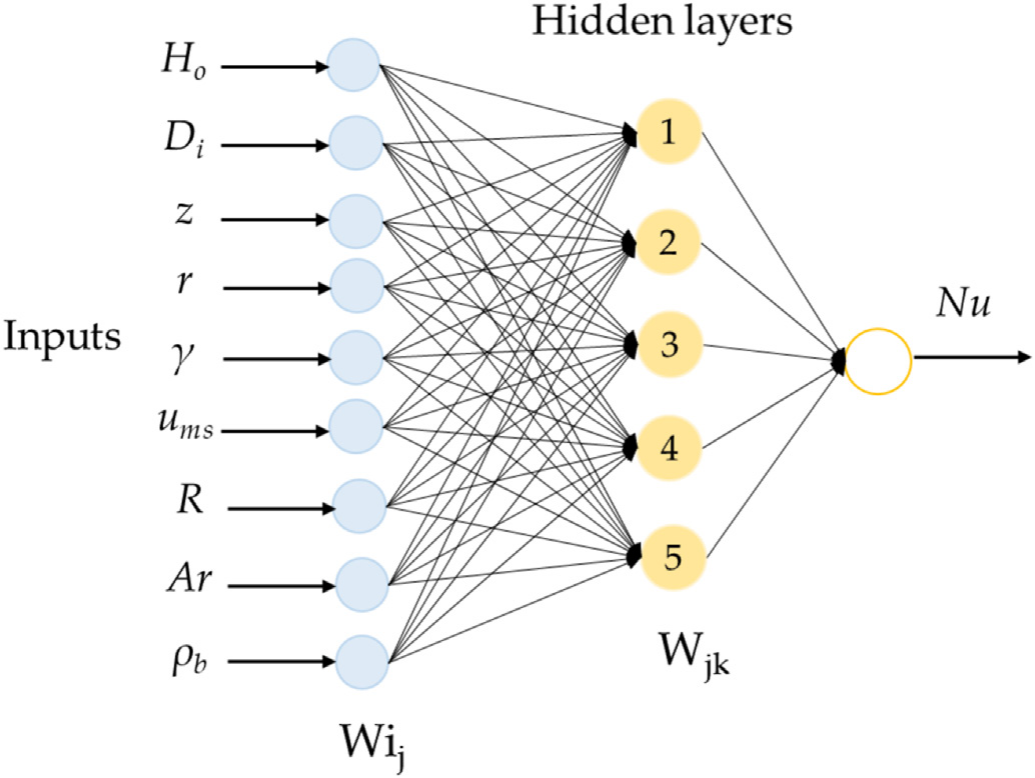
Schematic diagram of the multilayer single-output ANN for the dimensionless moduli Nusselt.

In this work, 162 different ANNs were tested, where the number of neurons in the hidden layer was iterated between 3 and 20, and the number of epochs was iterated between 10 and 200 in intervals of 10. The performance criteria to select the best configuration were based on the MSE and the correlation coefficient among the outputs and the targets of the tested ANN. When 60% of the data were used for training and the remaining 40% for testing, on an error estimation of 0.05, and using Eq. (1), it was discovered that five neurons are sufficient for the hidden layer in this study. Figure 2 illustrates the schematic of the proposed MLP neural network, including the nine neurons of the input layer and the five neurons of the hidden layer.

**Figure 3:**
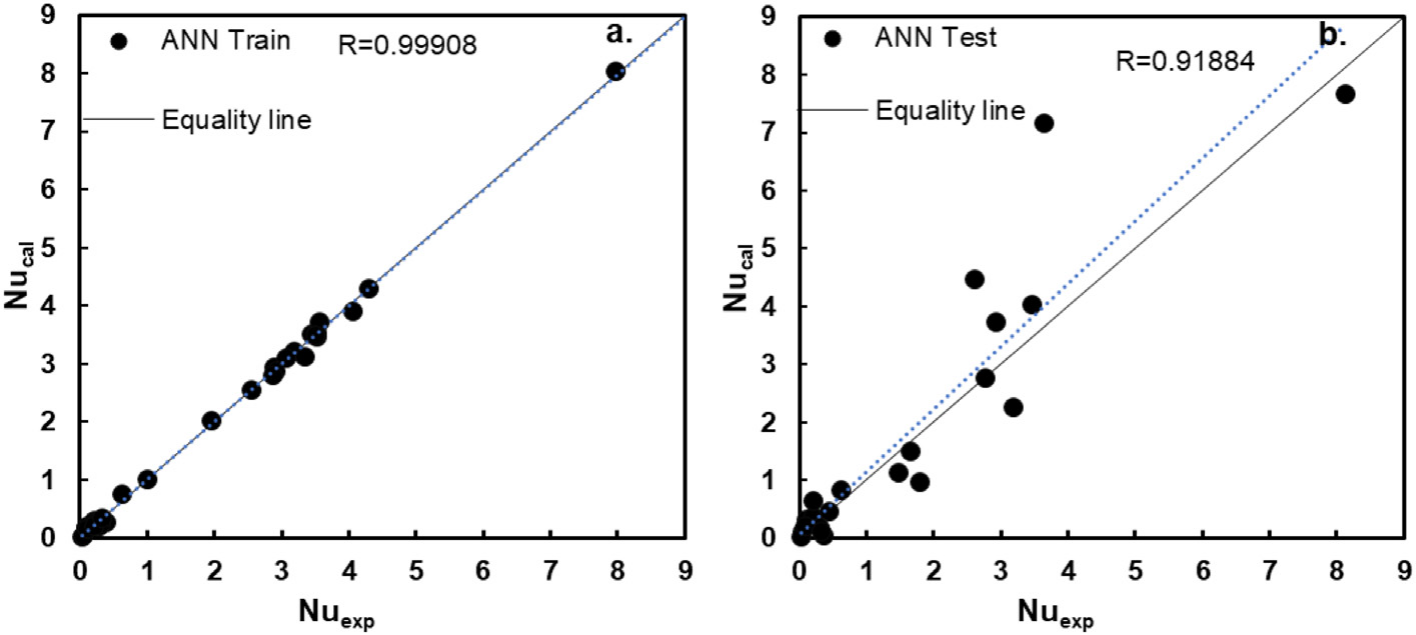
The regression plots for the model predicting dimensionless moduli Nusselt. a. Training data, b. Testing data.

The regression figures for the results of ANN to predict the dimensionless moduli of Nusselt are shown in Figure 3. The regression plots show the outputs predicted in the training and testing process versus the experimental targets or goals for the ANN. In the ideal case, the outputs are identical to the goals of the ANN. The regression plot should have a slope of 1 and a y-intersection of 0, with a coefficient of correlation of 1 for an ideal fit. In Figure 3, the ideal case is portrayed with a solid black line, and the regression line is portrayed as a blue line, which shows almost a perfect fit for the training data. Figure 3a shows a coefficient of correlation of 0.999, a slope of 0.997, and a bias very close to zero. For the test data in Figure 3b a lack of precision caused the regression plots to deviate from the ideal ones. However, the coefficient of correlation is still very close to one, with a value of 0.919, a slope of 1.086, and a bias near to zero. Furthermore, the regression values close to 1 indicate suitable fits.

**Figure 4:**
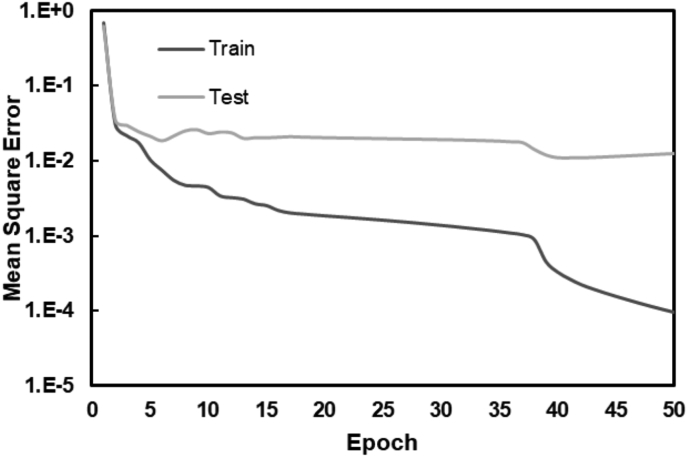
MSE versus the number of epochs in the hidden layer selected.

The training algorithm used with MATLAB attempts to decrease the MSE value between the outputs and goals in an iterative process. Figure 4 illustrates the convergence of the test operation, showing the MSE against the epoch number for the optimized ANN model. It was observed that the training and testing process was stopped after 50 epochs. The conversion of the MSE occurs at the lowest values in the training process (Figure 4). Similarly, the MSE converges in the testing process, to higher values. This difference may be since the ANNs are trained by the training data sets while they are evaluated in the test phase by untrained data sets. It is then that the training process generally leaves MSEs better than the testing processes. According to the results presented in Figure, the MSE of the training process has converged to 9.45·10^−5^. On the other hand, the MSE of the test process has converged 1.23·10^−2^ which indicates acceptable values for the trained test process and acceptable differences between the training and test MSE convergences.

According to the results obtained with the performance parameters, it can be concluded that the model obtained with the selected ANN configuration is adequate and can be used to predict the Nusselt value. The desire model obtained is described by the weights and biases for the ANN with the five neurons in the hidden layer are:

$$IW=\left[\begin{array}{c} \;\\ \;1.15\\ \;0.31\\ \;0.74\\ -0.74\\ -0.13 \end{array}\;\begin{array}{c} \;\\ \;2.84\\ \;1.13\\ -2.51\\ \;0.18\\ \;0.28 \end{array}\;\begin{array}{c} \;\\ \;3.99\\ -1.08\\ -2.09\\ -2.24\\ -0.28 \end{array}\;\begin{array}{c} \;\\ -3.70\\ \;0.91\\ -3.70\\ \;0.30\\ -1.87 \end{array}\;\begin{array}{c} \;\\ \;0.61\\ -0.05\\ \;0.12\\ -0.05\\ -0.28 \end{array}\;\begin{array}{c} \;\\ -0.44\\ \;0.86\\ \;1.84\\ -1.61\\ -0.63 \end{array}\;\begin{array}{c} \;\\ \;1.92\\ -0.13\\ -1.48\\ \;1.62\\ -0.63 \end{array}\;\begin{array}{c} \;\\ -0.08\\ -1.65\\ -0.96\\ \;0.32\\ \;0.17 \end{array}\right]\;b_{1}=\left[\begin{array}{c} -4.93\\ \;2.77\\ \;2.04\\ -2.66\\ \;2.53 \end{array}\right]$$

$$LW=\left[-1.41-2.01-1.40-2.56\;0.20\right]\;b_{2}=\left[-1.69\right]$$

where *IW* is the connection matrix between the input and hidden layer with *b*_*1*_ as the bias vector, and *LW* is the connection matrix between hidden layer and the output value with a bias of *b*_*2.*_

**Figure 5:**
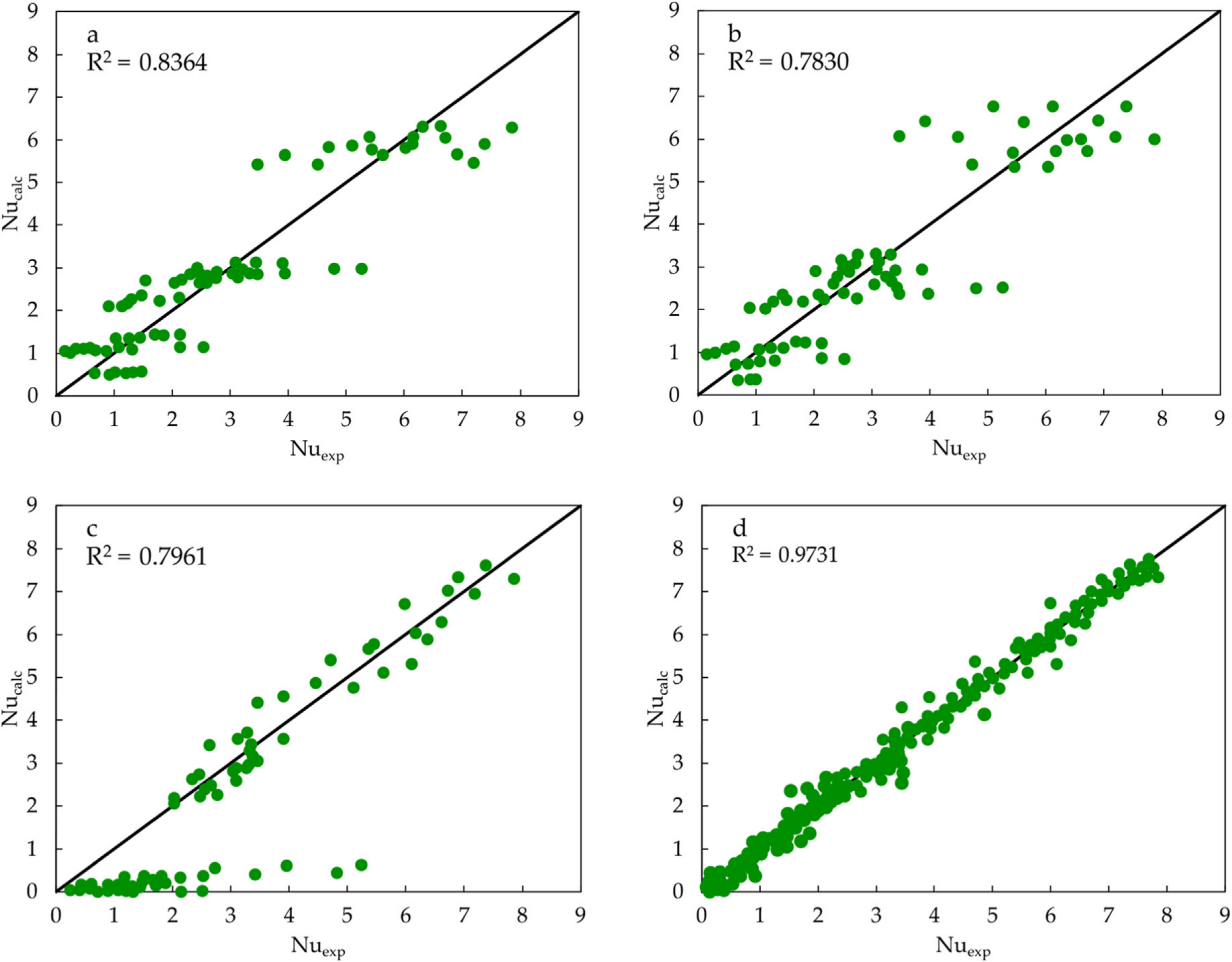
Comparison of Nu calculated by the available empirical models a. Kmiec [21], b. Kmiec [31], c. Kmiec and Kucharski [23], d. Saldarriaga et al. [14].

To support the applicability and superiority of the proposed model in this study, the ANN model was compared with the traditional model. The existing prediction models of heat transfer rate selected are the four empirical models presented in Table.1, which originated from the regression of the experimental data for the specific configuration of the CSBR, and the results are shown in Figure 5. It is evident that the proposed ANN model yields better prediction results with the measured values as compared to those calculated from empirical models. The predicted results of Figure 5 in the four evaluated models have a correlation coefficient of 0.8364 (Figure 5a), 0.7830 (Figure 5b), 0.7961 (Figure 5c), and 0.9731 (Figure 5d). It is observed that the overall fit obtained by the empirical models proposed by Kmiec and Jabarin [23] is poor, obtaining stratification of the predicted values for the cases of Kmiec [21] and Kmiec [31], and underestimation in the case of Kmiec and Kucharski [23], which validates that the models possess an obvious characteristic of low accuracy. However, as observed, there is a satisfactory fit for Figure 5d. This is because the empirical model used to construct this regression is the one proposed by Saldarriaga et al. [14], in which the value of *k* in Eq. (6) depends on the surface evaluated with a value for the bed surface and a different value for the bed. In contrast, the ANN models possess better prediction performance compared to the empirical models.

## 4 Conclusions

With these results, it is again demonstrated that neural networks play an important role when predicting fluid dynamic behaviors. It has been verified that the adjustment obtained by the correlation proposed by Saldarriaga et al. [14] is very good, showing that ANN models have superior prediction performance than the empirical correlations proposed in the literature. With this work, it is verified that the correlations proposed by Saldarriaga et al. [14] are the most suitable for predicting heat transfer in conical spouted bed reactors in the treatment of different biomasses. This is since both the surface and the bed are being considered, and this means that when dead zones are created inside the reactor, it can be predicted from these ANN models that the operation of the conical spouted bed reactor can be improved. Heat transfer in conical spouted bed reactors is an important factor in their scaling and operation. In the case of biomass, this plays an important role due to the geometry of the particles and their density. So, with what these ANN models turn out to be promising in the processes of scaling and industrial operation of the technology, with the good fit obtained in this work.

## Declarations

### Author contribution statement

Juan F. Saldarriaga, Ph.D.: Conceived and designed the experiments; Performed the experiments; Analyzed and interpreted the data; Contributed reagents, materials, analysis tools or data; Wrote the paper.

### Funding statement

This work was supported by the Dept. Civil and Environmental Engineering at Universidad de los Andes and the 10.13039/501100006070Universidad de los Andes the Early-Stage Research Found -FAPA- [P3.2017.3830], and thanks to the Masterʹs student Yuby Cruz graduate research assistant project [P3.2017.3830].

### Data availability statement

Data included in article/supp. material/referenced in article.

### Declaration of interest’s statement

The authors declare no conflict of interest.

### Additional information

No additional information is available for this paper.
